# Development, validation, and application of SYBR green-based qPCR assays for detection and quantification of *tetA, tetB*, and *tetO* genes in poultry and associated environments

**DOI:** 10.3389/fvets.2025.1754015

**Published:** 2026-01-16

**Authors:** Alessandra Piccirillo, Roberta Tolosi, Andrea Laconi

**Affiliations:** Department of Comparative Biomedicine and Food Science, University of Padua, Legnaro, Italy

**Keywords:** AMR, ARGs, qPCR, *tet* genes, *tetA*, *tetB*, *tetO*

## Abstract

**Introduction:**

Tetracycline resistance genes (*tet* genes) are among the most prevalent antimicrobial resistance determinants in poultry, raising concerns about their dissemination across animal, human, and environmental interfaces. This study aimed to develop and validate rapid, sensitive, and cost-effective SYBR Green-based quantitative PCR (qPCR) assays for the detection and quantification of *tetA, tetB*, and *tetO* genes in bacterial isolates and complex matrices.

**Methods:**

Following *in silico* and end-point PCR screening of ten published primer pairs, the most specific combinations were optimized for annealing temperature and primer concentration, and their analytical and diagnostic performances were evaluated.

**Results:**

The assays exhibited efficiencies of 80.7–93.6%, strong linearity (*R*^2^ > 0.99), and high repeatability (CV < 5%). Diagnostic sensitivity and specificity ranged from 92.11–100% and 91.38–100%, respectively. Application of the assays to fecal, cecal, and drinking water samples collected from free-range and short-chain poultry farms revealed that all but one samples were positive for at least one of the investigated *tet* genes, with prevalence ranging from 65.79% (*tetA*) to 93.33% (*tetO*).

**Discussion:**

These SYBR Green-based qPCR assays provide a robust, quantitative, and affordable tool for monitoring *tet* genes dissemination in poultry and associated environments. Their simplicity and reproducibility make them particularly suitable for large-scale surveillance programs and for use in settings where resources or access to probe-based platforms are limited.

## 1 Introduction

Tetracycline resistance genes (*tet* genes) are among the most widespread antimicrobial resistance determinants in livestock production (1). They occur in both pathogenic and commensal bacteria, raising concerns about their potential transfer to humans and the environment (2). Humans may be exposed to *tet*-carrying bacteria through the consumption of contaminated food (3), and antimicrobial resistance genes (ARGs) can also be exchanged between bacteria infecting animals and humans (4). In fact, *tet* genes are among the mobile ARGs shared between animal and human bacterial populations, with poultry identified as a major reservoir (4). Several studies have reported a high prevalence and diversity of *tet* genes in poultry worldwide (5–7). Among these, *tetA, tetB*, and *tetO* are the most frequently detected (5, 6, 8) and have been identified in bacterial species of both human and veterinary relevance, including *Escherichia coli, Enterococcus* spp., and *Campylobacter* spp. (7, 9, 10). The widespread occurrence of *tet* genes in poultry is likely associated with the use of tetracyclines, such as oxytetracycline, for treating respiratory and gastrointestinal infections (11). However, their detection even on antibiotic-free farms highlights their extensive dissemination and persistence in the environment (1). The rapid and reliable detection and quantification of key *tet* genes, such as *tetA, tetB*, and *tetO*, are essential for understanding their distribution and dissemination dynamics within poultry farms and throughout the poultry production chain. However, several recent studies still rely on conventional end-point PCR assays (6, 12), which are time-consuming and do not generate quantitative results. Quantitative data are, however, fundamental for characterizing ARGs dynamics and their dissemination patterns (13). In this study, we developed and validated three quantitative PCR (qPCR) assays based on SYBR Green paired with melting curve analysis for the detection and quantification of *tetA, tetB*, and *tetO* genes from environmental samples and complex matrices, such as feces and cecal content. To assess their effectiveness, these assays were applied to samples collected from free-range farms and from a short poultry production chain.

## 2 Materials and methods

### 2.1 Bacterial strains and DNA isolation

*E. coli* and *Campylobacter* spp. strains of poultry origin previously characterized by whole genome sequencing (14, 15) were used for primers selection, qPCR optimization, analytical and diagnostic validation, as well as positive control for the analysis of the field samples. The list of the strains used in this study, including the bacterial species, the resistance profile, the sequence accession numbers, and in which stage of the validation they were used, is reported in supplementary material 1. *E. coli* strains were resuscitated on nutrient agar (Microbiol, Italy) and incubated at 37 °C ± 5 °C for 24 ± 2 h. *Campylobacter* spp. strains were streaked on blood agar (Microbiol) and incubated at 41.5 °C ± 5 °C for 48 ± 2 h under a microaerophilic environment (CampyGen, Oxoid, UK). Bacterial genomic DNA (gDNA) was isolated using the Invisorb Spin Tissue Mini Kit (Invitek Molecular, Berlin, Germany) following the manufacturer's instruction. gDNA quantity was assessed using the Qubit dsDNA High Sensitivity kit (Thermo Fisher Scientific, Massachusetts, USA).

### 2.2 Primers selection and confirmation by end-point PCR

Based upon a thorough search of the literature, a total of ten primer pairs (Table 1) used for the detection of genes *tetA, tetB*, and *tetO* by end-point PCR were identified (5, 16, 17). Each primer pair was first checked *in silico* using primer BLAST (https://www.ncbi.nlm.nih.gov/tools/primer-blast/) and then tested for specificity against strains harboring *tetA* (*n* = 5), *tetB* (*n* = 5), and *tetO* (*n* = 6). To assess the risk of false positive results, each assay was also tested against the gDNA isolated from the bacteria not harboring their target gene. End-point PCRs were carried out in a total reaction volume of 25 μl, consisting of 12.5 μl DreamTaq PCR Master Mix 2X (Thermo Fisher Scientific), 0.5 μl of each primer at a concentration of 10 pmol/μl, and 5 ng of genomic DNA as the template. The thermal cycling program started with an initial denaturation at 95 °C for 2 min (min), followed by 35 cycles of denaturation at 95 °C for 30 s (s), annealing at 60 °C for 20 s, and extension at 72 °C for 20 s. A final extension step was performed at 72 °C for 7 min using an Applied Biosystems 2720 Thermal Cycler (Thermo Fisher Scientific). The resulting amplicons were analyzed by agarose gel electrophoresis on a 2% gel run at 100 V for 60 min. Selected PCR products were purified using the QIAquickPCR Purification Kit (Qiagen), following the manufacturer's instruction, and purified amplicons were Sanger sequenced by an external laboratory (BMR Genomics, Padua, Italy). Chromatograms were visualized and analyzed using BioEdit (v7.7.1). Edited sequences were screened in BLAST (https://blast.ncbi.nlm.nih.gov/Blast.cgi) for *tet* genes confirmation.

**Table 1:** List of the sequences of the primer pairs tested, the expected size of each PCR products, and their references.

**Primer pairs**		**Sequence 5^′^3^′^)**	**Expected size (bp)**	**Reference**
tetA1*	tet(A)_1F:	GCTACATCCTGCTTGCCTTC	210	(5)
	tet(A)_1R:	CATAGATCGCCGTGAAGAGG		
tetB1	tet(B)_1F:	TTGGTTAGGGGCAAGTTTTG	659	(5)
	tet(B)_1R:	GTAATGGGCCAATAACACCG		
tetO1	tet(O)_1F:	AACTTAGGCATTCTGGCTCAC	515	(5)
	tet(O)_1R:	TCCCACTGTTCCATATCGTCA		
tetA2	tet(A)_2F:	GCTGTTTGTTCTGCCGGAAA	Unknown	(17)
	tet(A)_2R:	GGTTAAGTTCCTTGAACGCAAACT		
tetB2	tet(B)_2F:	AGTGCGCTTTGGATGCTGTA	Unknown	(17)
	tet(B)_2R:	AGCCCCAGTAGCTCCTGTGA		
tetO2	tet(O)_2F:	ACGGAAAGTTTATTGTATACC	Unknown	(16)
	tet(O)_2R:	TGGCGTATCTATAATGTTGAC		
tetA3	tet(A)_3F:	CTCACCAGCCTGACCTCGAT	Unknown	(17)
	tet(A)_3R:	ACGTTGTTATAGAAGCCGCATAG		
tetB3*	tet(B)_3F:	GCCCAGTGCTGTTGTTGTCAT	Unknown	(17)
	tet(B)_3R:	TGAAAGCAAACGGCCTAAATACA		
tetO3*	tet(O)_3F:	ATGTGGATACTACAACGCATGAGATT	Unknown	(17)
	tet(O)_3R:	TGCCTCCACATGATATTTTTCCT		
tetO4	tet(O)_3F:	CAACATTAACGGAAAGTTTATTGTATACCA	Unknown	(17)
	tet(O)_3R:	TTGACGCTCCAAATTCATTGTATC		

* Primer pairs selected for the validation of the three assays.

### 2.3 Optimization of qPCR conditions of *tetA, tetB*, and *tetO* qPCR assays

After the initial selection, the optimization of qPCR conditions was carried out for primer pairs tetA1, tetB3, tetO2, and tetO3 (Table 1). All amplifications were carried out in a LightCycler^®^ 480 Roche (Basel, Switzerland) and using the PowerUp^TM^ SYBR^®^ Green Master Mix (Thermo Fisher Scientific). Different concentrations of each primer were tested for each primer pair (300/300 pmol/ml, 600/600 pmol/ml, 600/900 pmol/ml, 900/600 pmol/ml and 900/900 pmol/ml for forward and reverse primer respectively), using 2.5 μl of end-point PCR amplicons diluted to a final concentration of 0.002 ng/μl. The optimization steps were performed using the following amplification protocol: initial incubation at 50 °C for 2 min, followed by 2 min at 95 °C, and 45 cycles at 95 °C for 10 s and 50 °C−60 °C for 40 s. A melting curve between 40 °C and 95 °C was determined by adding a dissociation step after the last amplification cycle at a temperature transition rate of 4.4 °C/s. qPCR data analysis was performed using LightCycler^®^ 480 software version 1.5 (Roche). All qPCR reactions were performed in triplicate.

### 2.4 Analytical validation of *tetA, tetB*, and *tetO* qPCR assays

Standard curves were constructed to assess the efficiency and the dynamic range of each primer set using nine ten-fold dilutions prepared starting from an initial concentration of 0.002 ng/μl of end-point PCR amplicons. The assays' limit of detection (LoD) was considered as the lowest target amount detected in at least 50% of nine independent replicates tested by two operators in two independent runs. The repeatability of the three qPCRs was evaluated by two operators testing each three target dilutions, corresponding to high, medium, and low dilution (one dilution above the LoD), in triplicate during three independent experiments, at weekly intervals. Intra- and inter-run repeatability was assessed by calculating the coefficient of variation (CV). A %CV equal to 5% was adopted as cut-off value to assess the robustness of the assays. The specificity was assessed by testing the assays against the dilution panels not containing their respective target.

### 2.5 Diagnostic validation of *tetA, tetB*, and *tetO* qPCR assays

The diagnostic validation was carried out using a panel of 69 bacteria of known resistance profile (Supplementary Material 1). The panel was prepared by one operator and blindly tested by two other operators at weekly interval. Each sample was tested in triplicate. Samples with at least two replicates showing cycle threshold (Ct) values within the dynamic range of the assay and the expected melting temperature (Tm) were considered positive. The diagnostic sensitivity (DSe), the diagnostic specificity (DSp), and the overall accuracy (Acc) were calculated for each assay.

### 2.6 Identification and quantification of *tetA, tetB*, and *tetO* genes in field samples

The assays were used to assess the prevalence and to quantify their respective *tet* genes in 36 fecal, 11 cecal and 29 drinking water (DW) samples collected in previous studies (18, 19). Fecal, cecal, and DW samples were pre-treated as previously described (18, 19). Briefly, 25 g of feces were placed into a sterile 50-ml Falcon tube, mixed with 25 ml of phosphate-buffered saline (PBS), vortexed for 1 min, and centrifuged at 4,000 rpm for 10 min at 4 °C. DNA was then extracted from 250 mg of the resulting pellet using the DNeasy PowerLyzer PowerSoil^®^ kit (Qiagen, Hilden, Germany). Cecal contents were collected using sterile cotton swabs. Each swab was eluted in 1 mL of PBS, processed for 90 s at 25 Hz using a TissueLyser^®^ (Qiagen), the supernatant was recovered, and centrifuged at 4,000 rpm for 10 min at 4 °C. DNA was extracted from the resulting pellet using the DNeasy PowerSoil Pro^®^ Kit (Qiagen). Two liters of each water sample were filtered and DNA was extracted from the filter membranes using the PowerWater DNA kit (Qiagen). The list of the fields samples is reported in Supplementary Material 2. Field samples were tested in triplicates and samples with at least two replicates showing Ct values within the dynamic range of the assays and Tm matching the positive control were considered as positive. The 16S rRNA gene was also amplified (20) as internal process control and to normalize the target genes copy numbers. Positive (*tet*+) and negative controls (*tet*-) for each assay were included in each qPCR round.

### 2.7 Statistical and data analysis

Standard curves, assay efficiency, *R*^2^ values, DSe, DSp, overall accuracy, and repeatability were calculated using GraphPad Prism v10.5.0. Descriptive statistics for prevalence and gene quantification, as well as graphical representations of the results, were generated using the same software.

## 3 Results

### 3.1 Assays optimization

The optimal primers concentrations were established as 600/600 pmol/ml for *tetA* and as 300/300 pmol/ml for *tetB* and *tetO*. The annealing temperatures for maximizing assays' performances avoiding nonspecific amplification were defined as 55 °C for *tetA* and *tetB* and as 56 °C for *tetO*. The sequences of the selected primers are reported in Table 1.

### 3.2 Analytical and diagnostic performances of *tetA, tetB*, and *tetO* qPCR assays

**Figure 1:**
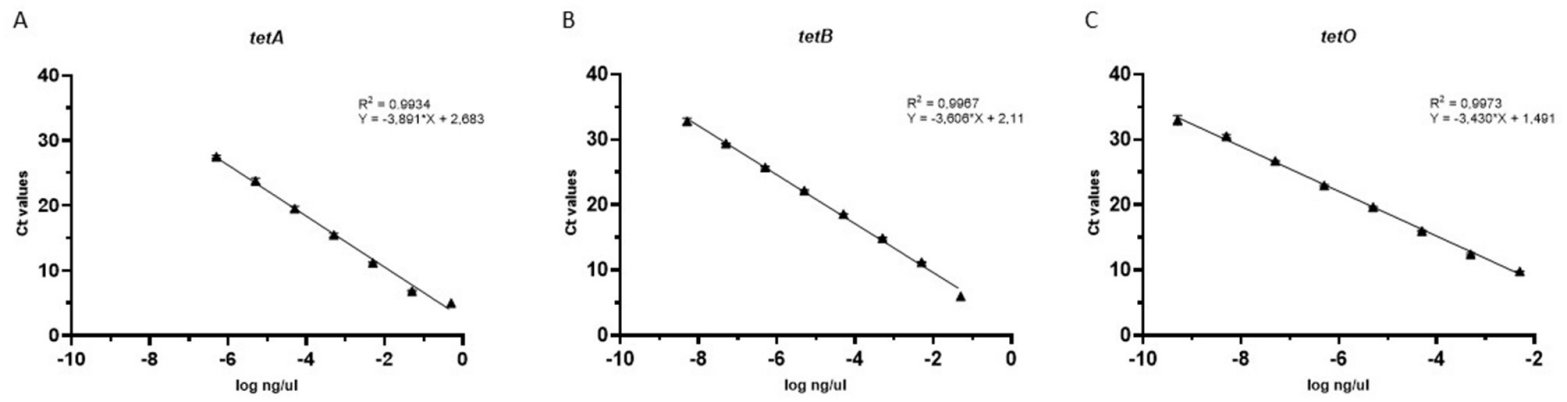
Standard curves for *tetA* **(A)**, *tetB* **(B)**, and *tetO* **(C)** assays. Each point of the curves represents the mean of three replicates; branches represent standard deviation. Linear and *R*^2^ are reported for each standard curve.

The qPCR assays exhibited efficiencies ranging from 80.7% for tetA to 93.6% for tetO, with all standard curves demonstrating excellent linearity (*R*^2^ > 0.99; Figure 1). The limits of quantification were determined to be 2,174.3, 39.7, and 3.8 gene copies per μl for the tetA, tetB, and tetO assays, respectively, while the corresponding limits of detection were 271.4, 4.0, and 3.8 gene copies per μl. All assays demonstrated high robustness and repeatability, as indicated by %CVs consistently below the adopted threshold (5%) both within and between runs (Table 2). DSe ranged from 92.11% for tetA to 100% for tetB and tetO, whereas DSp was assessed as 91.38%, 98.41%, and 100% for tetB, tetO, and tetA, respectively. The overall diagnostic accuracy was 95.71%, 92.86%, and 98.57% for tetA, tetB, and tetO, respectively.

**Table 2:** Repeatability of the qPCR assays for the detection and quantification of *tet* genes. Mean Ct (three replicates), standard deviation (SD) and coefficient of variation (CV) are reported for each assays according to target gene, dilution, operator and week of experiment.

***tet* gene**	**Plasmid copies number**	**Operator**	**Week 1**			**Week 2**			**Week 3**			**Total**		
			**Ct mean**	**SD**	**CV**	**Ct mean**	**SD**	**CV**	**Ct mean**	**SD**	**CV**	**Ct mean**	**SD**	**CV**
*tetA*	21,743,857.4	1	12.53	0.63	5.03	12.49	0.85	6.80	11.54	0.21	1.85	11.79	0.67	5.70
		2	11.43	0.31	2.68	11.64	0.20	1.74	11.13	0.14	1.27			
	217,438.6	1	20.70	0.52	2.51	19.36	0.16	0.82	19.36	0.26	1.35	19.55	0.68	3.49
		2	19.86	0.31	1.57	19.28	0.28	1.45	18.71	0.04	0.23			
	2,174.4	1	28.88	0.58	2.00	27.64	0.35	1.25	27.52	0.29	1.04	27.79	0.70	2.54
		2	27.83	0.44	1.57	27.35	0.03	0.11	27.58	0.23	0.84			
*tetB*	39,706,174.3	1	10.59	0.06	0.54	10.56	0.03	0.30	10.50	0.04	0.38	10.46	0.28	2.72
		2	10.78	0.03	0.25	9.90	0.05	0.48	10.42	0.08	0.73			
	39,706.2	1	21.49	0.02	0.09	21.58	0.02	0.08	21.64	0.06	0.30	21.48	0.32	1.48
		2	21.36	0.10	0.45	20.91	0.19	0.93	21.87	0.04	0.16			
	39.7	1	32.37	0.13	0.42	32.57	0.35	1.09	32.74	0.57	1.76	32.64	0.67	2.05
		2	32.95	0.14	0.41	31.93	0.30	0.93	33.57	0.16	0.48			
*tetO*	38,051,750.4	1	9.91	0.08	0.83	9.97	0.02	0.17	10.11	0.04	0.40	10.03	0.09	0.86
		2	10.03	0.08	0.75	10.06	0.01	0.10	10.12	0.03	0.29			
	38,051.8	1	20.80	0.06	0.29	20.37	0.11	0.54	20.56	0.03	0.15	20.64	0.28	1.33
		2	21.07	0.03	0.14	20.33	0.21	1.05	20.69	0.03	0.15			
	38.1	1	32.63	0.06	0.20	30.87	0.47	1.51	31.50	0.71	2.27	31.44	0.66	2.09
		2	31.77	0.61	1.91	31.02	0.43	1.40	31.13	0.11	0.35			

### 3.3 Detection and quantification of *tetA, tetB*, and *tetO* in field samples

**Figure 2:**
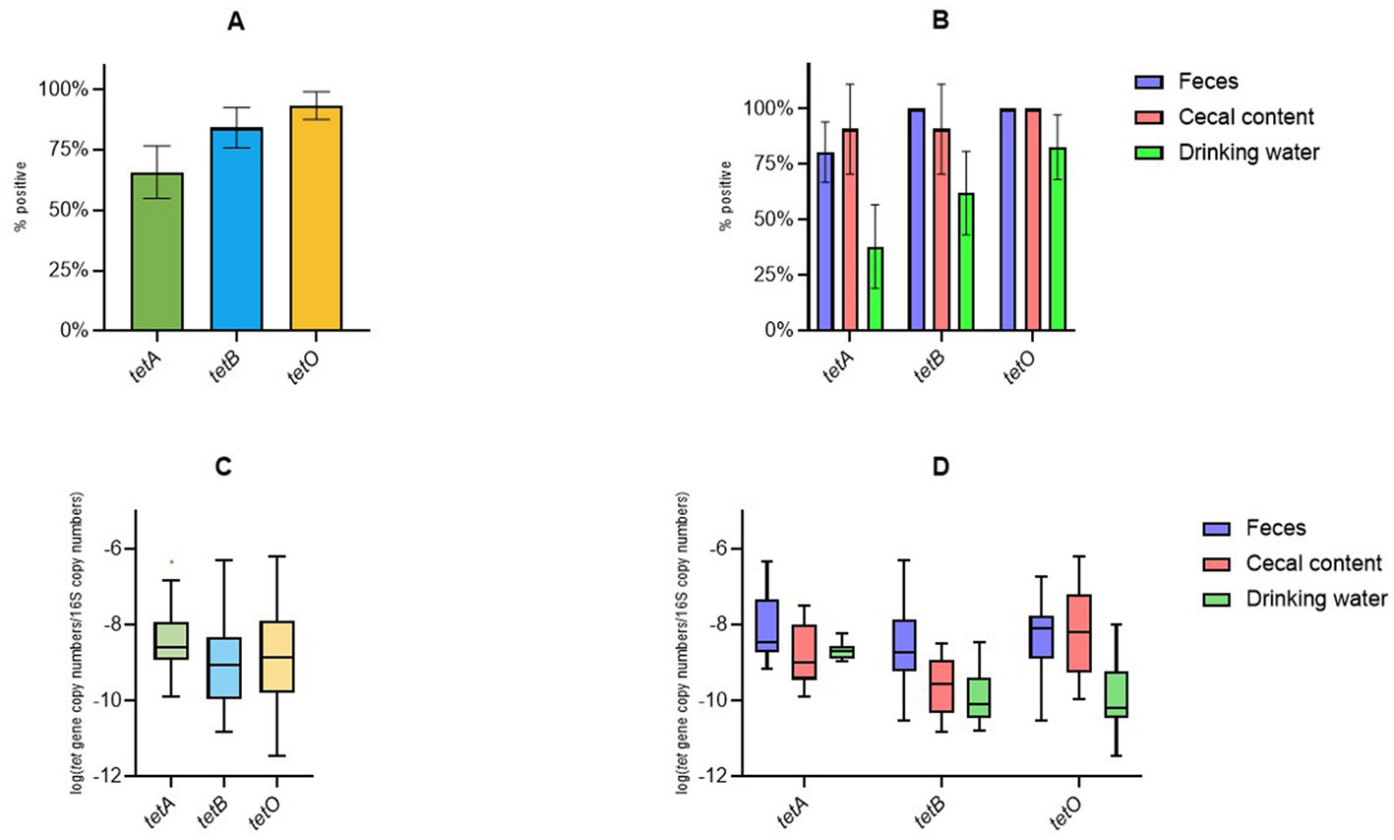
Prevalence and relative abundance of the investigated *tet* genes in fecal, cecal, and water samples. **(A)** Overall prevalence of *tet* genes across samples. **(B)** Prevalence according to the sample type. Whiskers represent 95% CI. Tukey box plots of the relative abundance of *tet* genes in all samples **(C)** and according to the sample type **(D)**.

With the exception of one water sample, all tested samples were positive for at least one of the investigated tet genes, with overall prevalence ranging from 65.79% (95% confidence interval (CI): 54.88%−76.70%) for tetA to 93.33% (95% CI: 87.56%−99.11%) for tetO (Figure 2A). The prevalence of tetA varied across sample types, from 37.93% (95% CI: 19.15%−56.71%) in drinking water to 90.91% (95% CI: 70.65%−100%) in cecal content (Figure 2B). For tetB, the lowest prevalence was observed in water samples (62.07%; 95% CI: 43.29%−80.85%), while all fecal samples tested positive (100%) (Figure 2B). Both fecal and cecal samples were positive for tetO, whereas its prevalence in drinking water was 82.76% (95% CI: 68.14%−97.38%) (Figure 2B). The mean relative abundance of the three genes, expressed as log10 (tet gene copy number/16S rRNA gene copy number), ranged from −8.36 for tetA to −9.03 for tetB (Figure 2C). Specifically, tetA abundance ranged from −8.75 in cecal content to −8.11 in fecal samples (Figure 2D). tetB abundance ranged from −9.84 in drinking water to −8.45 in feces (Figure 2D). tetO exhibited the highest relative abundance in cecal samples (−8.18) and the lowest in drinking water (−9.95) (Figure 2D).

## 4 Discussion

The main objective of the present study was to develop fast, sensitive, and reliable qPCR assays based on SYBR Green paired with melting curve analysis for the detection and quantification of key *tet* genes. Starting from previously published primer pairs (5, 16, 17), we identified the most effective pairs for each target gene, and then optimized and validated three assays that enables the rapid and confident detection and quantification of *tetA, tetB*, and *tetO* genes in poultry fecal and cecal samples, as well as in drinking water.

Over the past decade, several molecular methods, including end-point PCR (5), multiplex end-point PCR (12), probe-based qPCR (21), and loop-mediated isothermal amplification (LAMP) (22), have been developed for the detection of *tet* genes in bacterial isolates and/or complex samples (e.g., feces, soil, water). While these assays have proven sensitive and effective, each presents specific limitations. End-point PCR, for instance, is time-consuming and more prone to contamination, as it requires post-amplification electrophoresis for product visualization. Moreover, it does not provide quantitative results (5). Multiplex end-point PCR assays (6) allow simultaneous detection of multiple targets and thus reduce analysis time, yet they share the same drawbacks as conventional PCR. In addition, multiplex PCRs are more susceptible to misinterpretation when amplicons of similar size are generated and are prone to primer-dimer formation, which can lead to false-positive results (23). LAMP assays have been developed for only a few *tet* genes (i.e, *tetM* and *tetX*) (22, 24). However, these assays have mainly been validated on bacterial isolates, and their performance on field or clinical samples remains unclear. While LAMP may represent a cost-effective option for *tet* gene detection due to its minimal equipment and reagent requirements (25), these assays are qualitative rather than quantitative and require meticulous design to avoid non-specific amplification, a common cause of false positives (26). The use of labeled primers may reduce this issue but increases both cost and design complexity (27). Similarly, probe-based qPCR assays provide high specificity and sensitivity but require careful optimization of probe sequence, length, annealing temperature, and GC content to minimize cross-reactivity (28, 29). In addition, probes remain costly and sensitive to storage conditions, which may limit their use in low-resource settings (30, 31). In recent years, numerous studies have focused on developing more cost-effective alternatives to probe-based qPCR assays, with SYBR Green-based methods being the most extensively explored and developed (32, 33). Accordingly, the SYBR Green based qPCR assays developed and validated in this study offer a time- and cost-efficient alternative. These assays combine the speed and quantitative capability of qPCR with the simplicity and affordability of conventional PCR, making them suitable for detecting *tet* genes in both bacterial isolates and complex matrices. Owing to their robustness, sensitivity, and lower operational cost, these assays represent an ideal tool for large-scale surveillance and research applications, particularly in contexts where rapid and affordable detection of antimicrobial resistance determinants is essential. Additionally, due their cost-effectiveness the three assays may be easily employed in developing countries, where financial limitations can hamper monitoring antimicrobial resistance (34). The newly validated assays were successfully applied to detect and quantify *tetA, tetB*, and *tetO* genes in samples of both environmental (drinking water) and animal origin (feces and cecal content). The high prevalence of *tet* genes in fecal samples from free-range Italian poultry farms is consistent with previous reports (6) and reflects their widespread dissemination in the poultry sector worldwide (8, 35, 36).

In conclusion, the SYBR Green-based qPCR assays developed and validated in this study provide a rapid, sensitive, and cost-effective tool for the detection and quantification of *tetA, tetB*, and *tetO* genes in both bacterial isolates and complex matrices. Compared with previously available molecular methods, these assays offer an optimal balance of analytical performance, affordability, and operational simplicity. This makes them particularly suitable for large-scale monitoring of antimicrobial resistance in both research and diagnostic laboratories. Their successful application to samples of animal and environmental origin further demonstrates their versatility and robustness under field conditions. By enabling accurate quantification of key tetracycline resistance determinants, these assays provide a valuable resource for surveillance programs and risk assessment studies. Future research should focus on optimizing and developing SYBR Green-based qPCR assays targeting additional key and emerging *tet* resistance genes. Such assays will support efforts to understand and mitigate the spread of antimicrobial resistance within poultry production systems and beyond, from a One Health perspective.

## Data Availability

The original contributions presented in the study are included in the article/Supplementary material, further inquiries can be directed to the corresponding author.
