# Correction: Development, validation, and application of SYBR green-based qPCR assays for detection and quantification of *tetA, tetB*, and *tetO* genes in poultry and associated environments

**DOI:** 10.3389/fvets.2026.1823649

**Published:** 2026-03-19

**Authors:** Alessandra Piccirillo, Roberta Tolosi, Andrea Laconi

**Affiliations:** Department of Comparative Biomedicine and Food Science, University of Padua, Legnaro, Italy

**Keywords:** AMR, ARGs, qPCR, *tet* genes, *tetA*, *tetB*, *tetO*

In the published article there was a mistake in **Acknowledgments**, where the incorrect surname “Carollo” was used.

The correct statement reads:

“The Authors would like to thank Dr. Claudia Chirollo, Dr. Cristiana Penon, and Dr. Francesco Galuppo for the support during the sampling phase and Ms. Giulia Gagetta for her support during the validation phase.”

The original version of this article has been updated.

