# Supplementary material for: Development, validation, and application of SYBR green-based qPCR assays for detection and quantification of tetA, tetB, and tetO genes in poultry and associated environments
Source: Front Vet Sci. 2026 Jan 16;12:1754015. doi: 10.3389/fvets.2025.1754015 (PMC12856918; doi:10.3389/fvets.2025.1754015)
Supplement: Supplementary file 1 [file Table_1.docx]

Supplementary Material

# Supplementary Table 1. List of bacterial strains used for the optimization and validation of the three real-time qPCR assays. For each strain, the table reports the strain ID, species, *tet* gene carried, accession number, and the experimental step in which it was used.

| strain_id | Species | *tet* gene (WGS) | Accession number | Optimization and validation steps | | | |
| --- | --- | --- | --- | --- | --- | --- | --- |
|  |  |  |  | **Primers selection** | **qPCR Optimization** | **Analitical validation** | **Diagnostic validation** |
| EC-1 | *Escherichia coli* | *tetA* | ESC_MA5046AA | X |  |  | X |
| EC-2 | *Escherichia coli* | *tetA* | ESC_MA5620AA | X | X | X | X |
| EC-3 | *Escherichia coli* | *tetA* | ESC_MA5033AA | X |  |  | X |
| EC-4 | *Escherichia coli* | *tetA* | ESC_MA5637AA |  |  |  | X |
| EC-6 | *Escherichia coli* | *tetA* | ESC_MA5047AA |  |  |  | X |
| EC-7 | *Escherichia coli* | *tetA* | ESC_MA5036AA |  |  |  | X |
| EC-8 | *Escherichia coli* | *tetA* | ESC_MA5037AA |  |  |  | X |
| EC-10 | *Escherichia coli* | *tetA* | ESC_MA5594AA |  |  |  | X |
| EC-11 | *Escherichia coli* | *tetA* | ESC_MA5600AA |  |  |  | X |
| EC-12 | *Escherichia coli* | *tetA* | ESC_MA4974AA |  |  |  | X |
| EC-13 | *Escherichia coli* | None | ESC_MA5609AA |  |  |  | X |
| EC-14 | *Escherichia coli* | *tetA* | ESC_MA5608AA |  |  |  | X |
| EC-16 | *Escherichia coli* | *tetA* | ESC_MA5618AA |  |  |  | X |
| EC-19 | *Escherichia coli* | None | ESC_MA5049AA |  |  |  | X |
| EC-21 | *Escherichia coli* | *tetA* | ESC_MA5625AA |  |  |  | X |
| EC-22 | *Escherichia coli* | *tetA* and *tetB* | ESC_MA5622AA | X | X | X | X |
| EC-26 | *Escherichia coli* | *tetA* | ESC_MA5624AA |  |  |  | X |
| EC-27 | *Escherichia coli* | *tetA* | ESC_MA5631AA |  |  |  | X |
| EC-32 | *Escherichia coli* | *tetA* | ESC_MA5628AA |  |  |  | X |
| EC-33 | *Escherichia coli* | None | ESC_MA5629AA |  |  |  | X |
| EC-34 | *Escherichia coli* | None | ESC_MA5632AA |  |  |  | X |
| EC-43 | *Escherichia coli* | *tetA* | ESC_MA5639AA |  |  |  | X |
| EC-44 | *Escherichia coli* | *tetA* | ESC_MA5640AA |  |  |  | X |
| EC-45 | *Escherichia coli* | *tetA* | ESC_MA5642AA |  |  |  | X |
| EC-55 | *Escherichia coli* | *tetA* | ESC_MA5649AA |  |  |  | X |
| EC-56 | *Escherichia coli* | *tetA* | ESC_MA5650AA |  |  |  | X |
| EC-57 | *Escherichia coli* | *tetA* | ESC_MA5651AA |  |  |  | X |
| EC-58 | *Escherichia coli* | *tetB* | ESC_MA5652AA | X | X | X | X |
| EC-59 | *Escherichia coli* | None | ESC_MA5653AA |  |  |  | X |
| EC-62 | *Escherichia coli* | *tetB* | ESC_MA5041AA |  |  |  | X |
| EC-69 | *Escherichia coli* | *tetB* | ESC_MA5024AA | X |  |  | X |
| EC-70 | *Escherichia coli* | *tetB* | ESC_MA5042AA | X |  |  | X |
| EC-73 | *Escherichia coli* | None | ESC_MA5655AA |  |  |  | X |
| EC-74 | *Escherichia coli* | *tetB* | ESC_MA5040AA |  |  |  | X |
| EC-79 | *Escherichia coli* | *tetB* | ESC_MA5026AA |  |  |  | X |
| EC-80 | *Escherichia coli* | None | ESC_MA5039AA |  |  |  | X |
| EC-82 | *Escherichia coli* | *tetB* | ESC_MA5048AA |  |  |  | X |
| EC-84 | *Escherichia coli* | *tetB* | ESC_MA5038AA |  |  |  | X |
| EC-99 | *Escherichia coli* | None | ESC_MA5029AA |  |  |  | X |
| EC-101 | *Escherichia coli* | *tetA* and *tetB* | ESC_MA5593AA | X |  |  | X |
| EC-104 | *Escherichia coli* | None | ESC_MA5595AA |  |  |  | X |
| EC-117 | *Escherichia coli* | None | ESC_MA5604AA |  |  |  | X |
| EC-119 | *Escherichia coli* | *tetB* | ESC_MA5606AA |  |  |  | X |
| EC-127 | *Escherichia coli* | None | ESC_MA5050AA |  |  |  | X |
| EC-132 | *Escherichia coli* | None | ESC_MA5051AA |  |  |  | X |
| EC-133 | *Escherichia coli* | *tetB* | ESC_MA4987AA |  |  |  | X |
| EC-137 | *Escherichia coli* | None | ESC_MA4988AA |  |  |  | X |
| EC-146 | *Escherichia coli* | None | ESC_MA5611AA |  |  |  | X |
| Ca_02 | *Campylobacter coli* | *tetO* | JAAQZJ000000000 | X |  |  | X |
| Ca_09 | *Campylobacter jejuni* | *tetO* | JAAQZK000000000 | X | X | X | X |
| Ca_10 | *Campylobacter jejuni* | *tetO* | JAAQZL000000000 |  |  |  | X |
| Ca_12 | *Campylobacter coli* | *tetO* | JAAQZN000000000 | X |  |  | X |
| Ca_14 | *Campylobacter jejuni* | *tetO* | JAAQZP000000000 |  |  |  | X |
| Ca_15 | *Campylobacter coli* | *tetO* | JAAQZQ000000000 |  |  |  | X |
| Ca_18 | *Campylobacter jejuni* | *tetO* | JAAQZT000000000 | X |  |  | X |
| Ca_19 | *Campylobacter jejuni* | *tetO* | JAAQZU000000000 | X |  |  | X |
| Ca_27 | *Campylobacter coli* | *tetO* | JAAQZW000000000 | X |  |  | X |

**Supplementary Table 2.** List of field samples. For each sample, the table reports the sample type, time-point, location, year of isolation, and its reference.

| New ID | Sample type | Time-point | Location | Year of Sampling | Reference |
| --- | --- | --- | --- | --- | --- |
| SFC_1 | Faeces | Before slaughtering | Litter | 2024 | Laconi et al., 2025 |
| SFC_2 | Faeces | Before slaughtering | Litter | 2024 | Laconi et al., 2025 |
| SFC_3 | Faeces | Before slaughtering | Litter | 2024 | Laconi et al., 2025 |
| SFC_4 | Faeces | Before slaughtering | Litter | 2024 | Laconi et al., 2025 |
| SFC_5 | Faeces | Before slaughtering | Litter | 2024 | Laconi et al., 2025 |
| SFC_6 | Faeces | Before slaughtering | Litter | 2024 | Laconi et al., 2025 |
| SFC_7 | Faeces | Before slaughtering | Litter | 2024 | Laconi et al., 2025 |
| SFC_8 | Faeces | Before slaughtering | Litter | 2024 | Laconi et al., 2025 |
| SFC_9 | Faeces | Before slaughtering | Litter | 2024 | Laconi et al., 2025 |
| SFC_10 | Faeces | Before slaughtering | Litter | 2024 | Laconi et al., 2025 |
| SFC_11 | Faeces | Before slaughtering | Litter | 2024 | Laconi et al., 2025 |
| SFC_12 | Intestine | At slaughtering | Carcass | 2024 | Laconi et al., 2025 |
| SFC_13 | Intestine | At slaughtering | Carcass | 2024 | Laconi et al., 2025 |
| SFC_14 | Intestine | At slaughtering | Carcass | 2024 | Laconi et al., 2025 |
| SFC_15 | Intestine | At slaughtering | Carcass | 2024 | Laconi et al., 2025 |
| SFC_16 | Intestine | At slaughtering | Carcass | 2024 | Laconi et al., 2025 |
| SFC_17 | Intestine | At slaughtering | Carcass | 2024 | Laconi et al., 2025 |
| SFC_18 | Intestine | At slaughtering | Carcass | 2024 | Laconi et al., 2025 |
| SFC_19 | Intestine | At slaughtering | Carcass | 2024 | Laconi et al., 2025 |
| SFC_20 | Intestine | At slaughtering | Carcass | 2024 | Laconi et al., 2025 |
| SFC_21 | Intestine | At slaughtering | Carcass | 2024 | Laconi et al., 2025 |
| SFC_22 | Intestine | At slaughtering | Carcass | 2024 | Laconi et al., 2025 |
| FR_1 | Faeces | Beginning of cycle | Litter | 2022 | Piccirillo et al., 2024 |
| FR_2 | Faeces | Beginning of cycle | Litter | 2022 | Piccirillo et al., 2024 |
| FR_3 | Faeces | Beginning of cycle | Litter | 2022 | Piccirillo et al., 2024 |
| FR_4 | Faeces | Beginning of cycle | Litter | 2022 | Piccirillo et al., 2024 |
| FR_5 | Faeces | Beginning of cycle | Litter | 2022 | Piccirillo et al., 2024 |
| FR_6 | Faeces | Beginning of cycle | Litter | 2022 | Piccirillo et al., 2024 |
| FR_7 | Faeces | Beginning of cycle | Litter | 2022 | Piccirillo et al., 2024 |
| FR_9 | Faeces | Beginning of cycle | Litter | 2022 | Piccirillo et al., 2024 |
| FR_10 | Faeces | Beginning of cycle | Litter | 2022 | Piccirillo et al., 2024 |
| FR_11 | Faeces | Beginning of cycle | Litter | 2022 | Piccirillo et al., 2024 |
| FR_12 | Faeces | Beginning of cycle | Litter | 2022 | Piccirillo et al., 2024 |
| FR_13 | Faeces | Beginning of cycle | Litter | 2022 | Piccirillo et al., 2024 |
| FR_14 | Faeces | Before slaughtering | Litter | 2022 | Piccirillo et al., 2024 |
| FR_15 | Faeces | Before slaughtering | Litter | 2022 | Piccirillo et al., 2024 |
| FR_16 | Faeces | Before slaughtering | Litter | 2022 | Piccirillo et al., 2024 |
| FR_17 | Faeces | Before slaughtering | Litter | 2022 | Piccirillo et al., 2024 |
| FR_18 | Faeces | Before slaughtering | Litter | 2022 | Piccirillo et al., 2024 |
| FR_19 | Faeces | Before slaughtering | Litter | 2022 | Piccirillo et al., 2024 |
| FR_20 | Faeces | Before slaughtering | Litter | 2022 | Piccirillo et al., 2024 |
| FR_21 | Faeces | Before slaughtering | Litter | 2022 | Piccirillo et al., 2024 |
| FR_22 | Faeces | Before slaughtering | Litter | 2022 | Piccirillo et al., 2024 |
| FR_23 | Faeces | Before slaughtering | Litter | 2022 | Piccirillo et al., 2024 |
| FR_24 | Faeces | Before slaughtering | Litter | 2022 | Piccirillo et al., 2024 |
| FR_25 | Faeces | Before slaughtering | Litter | 2022 | Piccirillo et al., 2024 |
| FR_26 | Faeces | Before slaughtering | Litter | 2022 | Piccirillo et al., 2024 |
| FR_27 | Water | Beginning of cycle | Water tank | 2022 | Piccirillo et al., 2024 |
| FR_28 | Water | Beginning of cycle | Water tank | 2022 | Piccirillo et al., 2024 |
| FR_29 | Water | Beginning of cycle | End of water line | 2022 | Piccirillo et al., 2024 |
| FR_30 | Water | Beginning of cycle | Water tank | 2022 | Piccirillo et al., 2024 |
| FR_31 | Water | Beginning of cycle | End of water line | 2022 | Piccirillo et al., 2024 |
| FR_32 | Water | Beginning of cycle | End of water line | 2022 | Piccirillo et al., 2024 |
| FR_33 | Water | Beginning of cycle | Water tank | 2022 | Piccirillo et al., 2024 |
| FR_34 | Water | Beginning of cycle | End of water line | 2022 | Piccirillo et al., 2024 |
| FR_35 | Water | Beginning of cycle | End of water line | 2022 | Piccirillo et al., 2024 |
| FR_36 | Water | Beginning of cycle | End of water line | 2022 | Piccirillo et al., 2024 |
| FR_37 | Water | Beginning of cycle | Water tank | 2022 | Piccirillo et al., 2024 |
| FR_38 | Water | Beginning of cycle | End of water line | 2022 | Piccirillo et al., 2024 |
| FR_39 | Water | Before slaughtering | End of water line | 2022 | Piccirillo et al., 2024 |
| FR_40 | Water | Before slaughtering | Water tank | 2022 | Piccirillo et al., 2024 |
| FR_41 | Water | Before slaughtering | End of water line | 2022 | Piccirillo et al., 2024 |
| FR_42 | Water | Before slaughtering | Water tank | 2022 | Piccirillo et al., 2024 |
| FR_43 | Water | Before slaughtering | End of water line | 2022 | Piccirillo et al., 2024 |
| FR_44 | Water | Before slaughtering | End of water line | 2022 | Piccirillo et al., 2024 |
| FR_45 | Water | Before slaughtering | Water tank | 2022 | Piccirillo et al., 2024 |
| FR_46 | Water | Before slaughtering | End of water line | 2022 | Piccirillo et al., 2024 |
| FR_47 | Water | Before slaughtering | Water tank | 2022 | Piccirillo et al., 2024 |
| FR_48 | Water | Before slaughtering | End of water line | 2022 | Piccirillo et al., 2024 |
| FR_49 | Water | Before slaughtering | End of water line | 2022 | Piccirillo et al., 2024 |
| FR_50 | Water | Before slaughtering | End of water line | 2022 | Piccirillo et al., 2024 |
| FR_51 | Water | Before slaughtering | Water tank | 2022 | Piccirillo et al., 2024 |
| FR_52 | Water | Before slaughtering | End of water line | 2022 | Piccirillo et al., 2024 |
| FR_53 | Water | Beginning of cycle | End of water line | 2022 | Piccirillo et al., 2024 |
| FR_54 | Water | Beginning of cycle | End of water line | 2022 | Piccirillo et al., 2024 |
| FR_55 | Water | Beginning of cycle | End of water line | 2022 | Piccirillo et al., 2024 |
